# Pathophysiology and transcriptomic analysis of *Picea koraiensis* inoculated by bark beetle-vectored fungus *Ophiostoma bicolor*

**DOI:** 10.3389/fpls.2022.944336

**Published:** 2022-07-19

**Authors:** Ya Liu, Qinzheng Zhou, Zheng Wang, Huiming Wang, Guiheng Zheng, Jiaping Zhao, Quan Lu

**Affiliations:** ^1^Key Laboratory of Forest Protection of National Forestry and Grassland Administration, Ecology and Nature Conservation Institute, Chinese Academy of Forestry, Beijing, China; ^2^State Key Laboratory of Tree Genetics and Breeding, Institute of Ecological Conservation and Restoration, Chinese Academy of Forestry, Beijing, China

**Keywords:** transcriptome, ophiostomatoid fungi, monoterpenoids, spruce, *Ips*

## Abstract

Ophiostomatoid fungi exhibit a complex relationship with bark beetles; exhausting of host tree defenses is traditionally regarded as one of the key benefits provided to beetle vectors. *Ophiostoma bicolor* is one of the dominant species of the mycobiota associated with *Ips* genus bark beetles which infect the spruce trees across the Eurasian continent. Host spruce trees resist fungal invasion through structural and inducible defenses, but the underlying mechanisms at the molecular level, particularly with respect to the interaction between bark beetle-associated fungi and host trees, remain unclear. The aim of this study was to observe the pathological physiology and molecular changes in *Picea koraiensis* seedlings after artificial inoculation with *O. bicolor* strains (TS, BH, QH, MX, and LWQ). This study showed that *O. bicolor* was a weakly virulent pathogen of spruce, and that the virulent of the five *O. bicolor* strains showed differentiation. All *O. bicolor* strains could induce monoterpenoid release. A positive correlation between fungal virulence and release of monoterpenoids was observed. Furthermore, the release rate of monoterpenoids peaked at 4 days post-inoculation (dpi) and then decreased from 4 to 90 dpi. Transcriptomic analysis at 4 dpi showed that many plant-pathogen interaction processes and mitogen-activated protein kinase (MAPK) metabolic processes were activated. The expression of monoterpenoid precursor synthesis genes and diterpenoid synthesis genes was upregulated, indicating that gene expression regulated the release rate of monoterpenoids at 4 dpi. The enriched pathways may reveal the immune response mechanism of spruce to ophiostomatoid fungi. The dominant *O. bicolor* possibly induces the host defense rather than defense depletion, which is likely the pattern conducted by the pioneers of beetle-associated mycobiota, such as *Endoconidiophora* spp.. Overall, these results facilitate a better understanding of the interaction mechanism between the dominant association of beetles and the host at the molecular level.

## Introduction

Spruce is an evergreen woody plant that is distributed in cold temperate and subalpine regions. The genus consists of approximately 50 species, including 20 species and five varieties found in China (Chen, 2013). In recent years, pest outbreaks have been accelerated by climate change and increasing international trade. Spruce forests in the Northern Hemisphere, especially in Europe, have been severely affected by bark beetles of genus *Ips* (Coleoptera: Curculionidae: Scolytinae) (Hlásny et al., 2021). Bark beetles and fungi have formed complex associations during long-term co-evolution, resulting in the ability for the beetles to carry ophiostomatoid fungi (Six, 2012). The combined impact of the association between beetle pests and ophiostomatoid fungi has been studied for a long time (Kirisits and Konrad, 2004; Wingfield et al., 2016) and has received attention in China since the turn of the century, in view of the successful invasion of *Dendroctonus valens* from North America and the epidemic outbreak of indigenous *Ips* spp. and *Dendroctonus armandii* (Lu et al., 2008; Sun et al., 2013; Wang et al., 2022).

Both beetle and fungal partners have developed from occasional and sporadic to relatively stable and intimate relationship during their historical co-occurrence in the same habits (Bleiker and Six, 2009; Biedermann and Vega, 2020; Chakraborty and Roy, 2021; Singh et al., 2021). The benefits of beetle vectors to fungi are evident; for example, beetles can bring the fungi to new hosts and create inoculation holes. However, the benefits of fungi to beetles are unclear and sometimes disputed (Six and Wingfield, 2011). Nevertheless, substantial evidences from studies in nutrition, chemical ecology, and molecular biology have supported that fungi (particularly ophiostomatoid fungi) can enhance the colonization success of beetles in the following ways: by producing ergosterol, which is indispensable for beetle development (Bentz and Six, 2006), improving food quality and availability (Davis et al., 2019; Dzurenko and Hulcr, 2022), detoxifying tree defense compounds (DiGuistini et al., 2011; Wadke et al., 2016; Itoh et al., 2018; Davis et al., 2019; Zhao et al., 2019a), attracting or repelling other individuals through semiochemicals (Zhao et al., 2015, 2019b; Kandasamy et al., 2019), or competitive exclusion of beetle pathogens (Davis et al., 2019). In addition, many members of the ophiostomatoid fungi are pathogens of forests and crops worldwide. Notorious diseases caused by ophiostomatoid fungi include Dutch elm disease, laurel wilt, and oak wilt on broadleaf trees, and stem canker stain and black root disease on coniferous trees, leading to severe ecological and economic losses (Henry, 1944; Brasier, 1986; Harrington et al., 2008; Santini and Faccoli, 2015). Artificial inoculation of certain ophiostomatoid fungi can generate inner bark necrosis and lead to sapwood blue staining and drying within a few months, severely hindering water transportation, affecting tree growth, and even causing the death of host plants (Yamaoka et al., 1998; Davydenko et al., 2017; Devkota and Eckhardt, 2018).

Conifers have evolved a combination of structural and inducible defenses against bark beetles and their associated fungi (Franceschi et al., 2005; Jones and Dangl, 2006; Celedon and Bohlmann, 2019). Studies on the chemical defense of conifers against bark beetle-ophiostomatoid fungal symbionts have shown that terpenes are toxic to the symbionts (Phillips and Croteau, 1999; Raffa et al., 2005; Keeling and Bohlmann, 2006; Pan et al., 2018; Celedon and Bohlmann, 2019; Ullah et al., 2021). The phloem tissue of *Pinus* spp. contains constitutive monoterpenes that provide immediate resistance to bark beetle attacks (Boone et al., 2011; Raffa et al., 2013); local concentrations of terpenes increase rapidly after beetle attack. When the induced terpene concentration levels exceed the beetle’s physiological tolerance threshold, they inhibit or repel beetles that arrive subsequently and alter the growth of fungi associated with beetles that are already present (Raffa et al., 2005; Erbilgin et al., 2017). *In vitro* experiments also confirmed that monoterpenes, such as pinene and limonene, can inhibit the growth of fungi (Zhao et al., 2019b; Fang et al., 2020; Wang et al., 2020a). Monoterpenes, such as α-pinene, with stronger growth inhibitory ability against fungi, have a stronger repelling effect on bark beetles (Fang et al., 2020). Long-term production of monoterpenoids consumes a large amount of carbon, which is essential for tree growth (Trapp and Croteau, 2001; Dai et al., 2022), impeding the growth of pest-infested trees. In addition to terpenes, phenolics seem less important in the induced defense against bark beetle-associated fungal infestation (Erbilgin et al., 2017).

RNA-seq facilitates genome-wide assessment of gene expression profiles in plants under various conditions, such as biotic and abiotic stresses (Conesa et al., 2016). At present, more and more studies have used this method to analyze host plant resistance (Nibedita and Jolly, 2017). In a transcriptomic comparison between healthy *Gastrodia elata* and those infected with *Penicillium oxalicum*, 10 potential resistance genes were identified, involving plant hormone signal transduction, jasmonic acid signaling, and plant-pathogen interaction pathways, revealing that the immune response mechanism of *G. elata* to fungal disease is a complex biological process (Wang et al., 2020b). In poplars infected with canker disease in the early stage, most of the carbon metabolism and transportation genes, aquaporin genes, and genes related to secondary metabolites and phenylpropane biosynthesis pathway were inhibited, while the expression of resistance genes was promoted (Li et al., 2019). The transcript abundance of Norway spruce leucoanthocyanidin reductase (LAR) genes increased significantly during *Endoconidiophora polonica* infection (Hammerbacher et al., 2014). Anthocyanin monomers can mitigate the effects of various abiotic stresses such as ultraviolet radiation and ozone by reducing oxidative stress and inhibiting bacterial growth and fungal spore germination (Jaakola et al., 2004; Karonen et al., 2006).

It is interesting to understand whether the beetle-vectored fungi cause plant disease and whether the plants develop resistance against them. *Ophiostoma bicolor* is one of the dominant associate fungi with *Ips* spp. and *Dendroctonus micans*, which attacked various spruce species in Europe and north China (Giordano et al., 2013; Nève Repe et al., 2018; Chang et al., 2019, 2020; Chakraborty et al., 2020; Wang et al., 2020c,^2021^). Previous studies have confirmed that the pioneer species in the associated fungal community of *Ips*, like *Endoconidiophora* spp., showed strong virulence, which could cause severe host necrosis and induce a drastic resistance response of host conifers. However, *O. bicolor* could cause a small amount of spruce sapwood blue-staining and drying, and form small necrotic lesions on the inner bark near the inoculation site, accompanied by bits of resin outflow, showing moderate to weak levels of virulence from a series of pathogenicity tests in Europe and Japan (Christiansen and Solheim, 1994; Yamaoka et al., 2000; Sallé et al., 2005; Repe et al., 2015). The pathogenicity of *O. bicolor* isolated from China is unknown, and whether *O. bicolor* can induce the host to produce a resistance response like the pioneer species need to be further investigated. In addition, the molecular mechanisms underlying host resistance to ophiostomatoid fungi remain unclear.

In this study, the representative strains of *O. bicolor* were inoculated on 4-year-old *Picea koraiensis* to observe the differences in virulence between various strains, given that different strains of the same pathogen species may have different levels of virulence (Lieutier et al., 2003; Sallé et al., 2005). Changes in host defense-related metabolites after inoculation were also analyzed and the defense mechanisms of spruce against *O. bicolor* were investigated at the molecular level.

## Materials and methods

### Plant materials and fungal strains

To compare the virulence of *O. bicolor* strains on spruces, 210 healthy 4-year-old *P. koraiensis* Nakai clonal seedlings were used in this study. These seedlings, which were 31–50 cm in height and 5.9–8.4 mm in diameter at the base of the plants, were grown outdoors in 20 cm pots containing a mixture of turfy soil and perlite (v:v = 4:1) in campus of Chinese Academy of Forestry in Beijing. The seedlings were well watered throughout the experiments.

Five *O. bicolor* strains, TS, BH, LWQ, QH, and MX were inoculated into the spruces. These strains were originally isolated from three *Ips* bark beetles (*Ips typographus* Linnaeus from northeast and northwest China, *Ips nitidus* Eggers from Qinghai-Tibet plateau, and *Ips hauseri* Reitter from northwest China) (Table 1) and their breeding galleries in infested spruces during the period from 2016 to 2018. Before inoculation, fungi were incubated at 25°C in darkness for 7 days.

**TABLE 1:** The information of *O. bicolor* isolate.

Strain no.	Host	Location	Beetle
TS	*Picea schrenkiana* Fischer Mey.	Urumqi, Xinjiang Province	*Ips hauseri Reitter*
BH	*P. koraiensis* Nakai	Erdao Baihe, Jilin Province	*I. typographus Linnaeus*
QH	*P. obovata* Ledeb.	Qinghe, Xinjiang Province	*I. typographus Linnaeus*
MX	*P. crassifolia* Kom.	Maixiu, Qinghai Province	*I. nitidus Eggers*
LWQ	*P. balfouriana* Rehd. et Wils	Riwoqê, Tibet	*I. nitidus Eggers*

### Treatments

On July 15, 2020, the seedlings were randomly assigned to seven different treatment groups with each treatment group including 30 replicates. Among them, five treatment groups were inoculated with TS, BH, LWQ, QH, and MX each, one was inoculated with 2% MEA without fungus, and another was a healthy control. Stems were drilled with a sterile 5 mm cork borer into the surface of xylem to create a small hole, and a 5 mm mycelium plug was placed in the hole using a sterile toothpick. The wound was covered with bark and wrapped with parafilm and tape to protect the wound against contamination and drying.

### Analysis of host monoterpenoids

At 4, 30, and 90 days post-inoculation (dpi), 10 seedlings per treatment were used to evaluate host response. The crown and stem of the seedlings were enclosed using a polyethylene (PE) film (48 cm × 60 cm; Reynolds Consumer Products, Lake Forest, IL, United States). Collection of volatile monoterpenoids from *P. koraiensis* was performed using the closed circulation dynamic headspace sampling system of Porapak-Q absorbent (i.e., 200 mg, 50–80 mesh, 0.6 mm diameter, 160 mm long glass tube, Merck KGaA, Darmstadt, Germany) through mini vacuum pumps (Atmospheric Sampling Instrument, QC-1S, Beijing Labor Protection Institute, China) at an airflow rate of 500 mL/min for 1 h. Sampling tubes containing volatiles trapped on the Porapak-Q absorbent were then sealed at both ends with aluminum foil and placed inside a warm box filled with dry ice. The host tree headspace volatiles were extracted from the sampling tubes with 2 ml of HPLC-grade n-hexane. Prior to gas chromatography (GC), each sample was concentrated to 50 μl with a mild nitrogen stream and stored at −20°C.

Sample analyses were performed using a GC equipped with a flame ionization detector (GC-FID) (Agilent Technologies, Palo Alto, CA, United States) and an automatic sampler for liquid sample injections to identify and quantify host monoterpenes. For each sample run, 1 μl of extract was injected into an HP-5 column (Agilent Technologies, 30 m × 0.25 mm i.d. × 0.25 μm film thickness). Analysis conditions were as follows: H_2_ as carrier gas at 15 psi column head pressure; flame ionization detector temperature 270°C, and injector temperature 250°C; the oven temperature program started at 45°C (kept isothermal, 1 min), and increased linearly to 105°C at 2°C min^–1^ (kept isothermal, 1 min), and then to 250°C at 15°C min^–1^ (kept isothermal, 30 min). Data acquisition and subsequent processing were performed using the Agilent ChemStation GC Systems software. Absolute amounts of monoterpenes were quantified according to the retention time using the following standards: (-)-α-pinene, camphene, β-pinene, myrcene, 3-carene, α-phellandrene, (-)-limonene, and γ-terpinene, and the monoterpene concentrations in the samples were calculated according to external standards.

### Analysis of fungal pathogenicity

At 30 and 90 dpi, 10 seedlings per treatment were used to evaluate fungal infection. All barks were removed from the lesion area around the inoculation point with a knife, and the length and width of each necrotic lesion were measured. Three random replicates of every treatment were selected, to take approximately 1 cm^2^ samples from the edges of each necrotic lesion for re-isolation of the fungi.

### Analysis of host transcriptome

The monoterpenoid release peak reached at 4 dpi and then distinctly decreased at later two sampling time points (30 and 90 dpi). Thus, the early molecular changes of *P. koraiensis* after infection by *O. bicolor* were analyzed. At 4 dpi, seedlings inoculated with the most and least virulent strains were used for transcriptome sequencing by Illumina. Phloem tissue was collected from a 1 × 4 cm square at the disease/health junction, wrapped in aluminum foil, and placed in dry ice. These samples were stored at −80°C in the laboratory prior to transcriptomic analysis.

Ribonucleic acid from each treatment group with three biological replicates was extracted using the RNAprep Pure Plant Kit (Polysaccharides and Polyphenolics-rich, TIANGEN, Beijing, China) by following the manufacturer’s protocol. RNA concentration and purity were measured using NanoDrop 2000 (Thermo Fisher Scientific, Wilmington, DE, United States). RNA integrity was assessed using the RNA Nano 6000 Assay Kit on the Agilent Bioanalyzer 2100 system (Agilent Technologies, Santa Clara, CA, United States). The obtained RNA was stored at −80°C.

The sequencing library was constructed using 1 μg RNA from each sample, which was generated using the NEBNext Ultra RN Library Prep Kit from Illumina (New England Biolabs, Ipswich, MA, United States) by following the manufacturer’s protocol. Briefly, mRNA was enriched using magnetic oligo (dT) beads. Fragmentation was carried out using divalent cations under elevated temperatures in NEBNext First Strand Synthesis Reaction Buffer (5X). The short mRNA fragments were used as templates, and the first-strand cDNA was synthesized using a random hexamer primer and M-MuLV reverse transcriptase. Buffer solution, dNTPs, RNase H, and DNA polymerase I were added to synthesize the second-strand cDNA. The remaining overhangs were converted into blunt ends via exonuclease/polymerase activity. After adenylation of the 3′ ends of the DNA fragments, NEBNext Adaptor with a hairpin loop structure was ligated for hybridization. The AMPure XP system (Beckman Coulter, Beverly, MA, United States) was then used for fragment size selection. Suitable fragments were used as templates for PCR amplification to generate a final cDNA library. Finally, the cDNA library was sequenced using the Illumina HiSeq™ 2000 system (Biomarker Technologies Co., Ltd., Beijing, China). The entire set of raw reads was submitted to the Gene Expression Omnibus (GEO) at NCBI under the accession number PRJNA835255.

To obtain high-quality reads to ensure the accuracy of the subsequent analyses, reads containing adapters and poly-N, and low-quality reads (reads containing more than 50% of bases with a *Q*-value of ≤ 10%) were removed from the sequencing results. Cleaned reads were then mapped to the reference genome of the Norway spruce using HISAT2 software (Kim et al., 2015). Mapped reads were assembled and quantified using the StringTie software (Pertea et al., 2015). The fragments per kilobase of transcript per million fragments mapped (FPKM) measure was used to estimate gene expression levels to determine significant changes in gene expression under different treatments. Hierarchical cluster analysis was used to evaluate the consistency of the sequencing data.

Gene functions were annotated based on the KEGG Ortholog database (KO) and Gene Ontology (GO) databases. Differential expression analysis was performed in the DESeq2 software (Love et al., 2014) for pairwise comparisons using a model based on a negative binomial distribution. The Benjamini–Hochberg method was used to control for the false discovery rate (FDR), with a *p*-value < 0.01. KOBAS software (Mao et al., 2005) was used to test the statistical enrichment of differentially expressed genes (DEGs) in KEGG pathways.

### Quantitative real-time PCR

The CFX96TM Real-Time system (Bio-Rad, Hercules, CA, United States) and SYBR Green FP205 Kit (TIANGEN, China) were used to quantify the expression levels of eight genes in the most and least virulent strain treatment and control. Primer 5.0 (Premier Biosoft) was used to design primer sequences (Supplementary Table 1), and the primers were synthesized by Sangon Biotech (Shanghai) Co., Ltd. Each sample contained three technical replicates. The relative expression levels were calculated using the 2^–ΔΔ^ *^Ct^* method and normalized using the actin gene (NCBI accession number: AAF03692) as the internal reference (OuYang et al., 2015).

### Data analysis

The area of the lesion was calculated by multiplying the length by the width (mm^2^) (Rajtar et al., 2021). The monoterpenoid release rate was analyzed based on the difference (Δ) between the treated and healthy groups. Transcriptomic analysis was performed using the BMK cloud platform.^1^ The lesion area generated by different strains in the same time period and monoterpenoid concentrations from different treatments at the same time or from same treatments at different times were analyzed by one-way analysis of variance (ANOVA). The relationship between the pathogenicity and monoterpenoid changes and the relationship between RNA-seq and quantitative reverse transcription PCR (RT-qPCR) were analyzed by Pearson correlation. IBM SPSS Statistics 19 was used to perform ANOVA (LSD test, α = 0.05) and Pearson correlation analysis.

## Results

### Lesion area and fungal re-isolation

**FIGURE 1:**
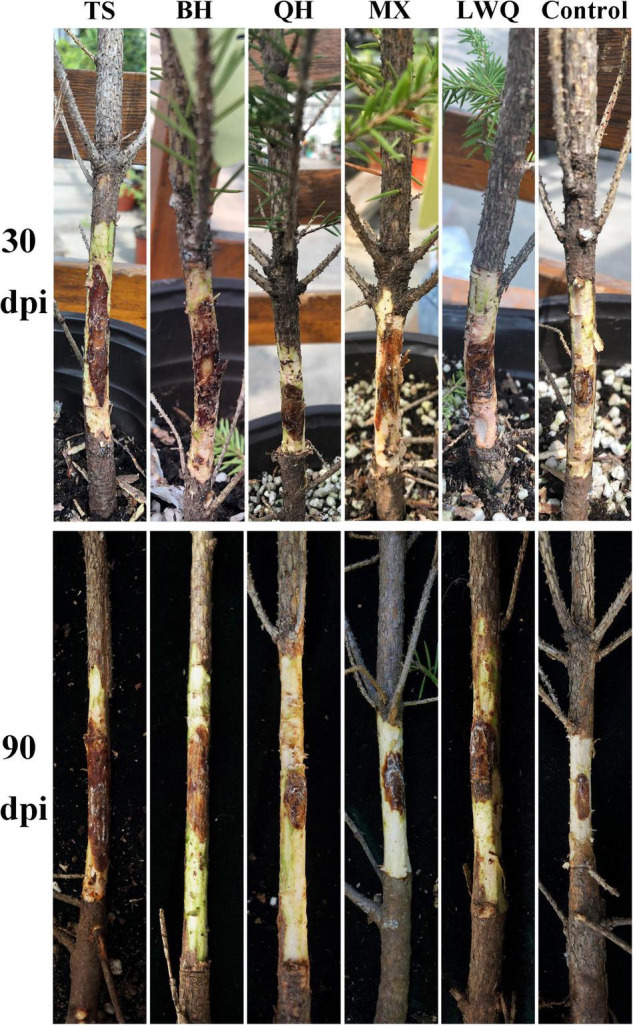
Disease development of *P. koraiensis* stems infected by *O. bicolor* at 30 and 90 dpi.

During the experiment, the inoculated *P. koraiensis* seedlings had no visible disease symptoms or wilt on the whole seedlings. After removal of the outer bark, all treatment seedlings inoculated with *O. bicolor* strains showed evident lesions on the phloem tissues of their stems (Figure 1).

As shown in Table 2 and Figure 1, at 30 dpi, the lesion area caused by TS was the largest among the five strains treatments (371.00 ± 37.45 mm^2^), followed by BH, QH, and MX. The lesion area caused by LWQ was the smallest (143.90 ± 30.94 mm^2^). ANOVA results showed that the lesion area of *O. bicolor* inoculation was significantly larger than that of the control (85.63 ± 10.00 mm^2^), and the lesion area caused by TS was significantly larger than that of other strains. At 90 dpi, the lesion area caused by TS was consistently the largest (432.72 ± 37.38 mm^2^) among the five strains treatments (Table 2; Figure 1), followed by BH, QH, and MX. The lesion area caused by LWQ was the smallest (160.53 ± 21.20 mm^2^). ANOVA results showed that the lesion area of *O. bicolor* inoculation was significantly larger than that of the control (93.06 ± 13.26 mm^2^), and the lesion area caused by LWQ was significantly smaller than that of other strains. The above results indicated that the pathogenicity of the five strains was differentiated, among which TS was the strongest, BH was the second, QH and MX were the third, and LWQ was the least. The mean re-isolation rate of the inoculated fungi from each of the three random lesion samples was 88.7%, confirming that necrotic lesions were caused by *O. bicolor* inoculation.

**TABLE 2:** The area of necrotic lesions caused by different *O. bicolor* isolate.

Strain no.	Mean lesion of inoculation (mm^2^)	
	30 dpi	90 dpi
TS	371.00 ± 37.45^a^	432.72 ± 37.38^a^
BH	255.56 ± 24.79^b^	316.11 ± 25.17^b^
QH	169.31 ± 20.3^c^	237.29 ± 32.28^bc^
MX	148.33 ± 17.4^c^	272.35 ± 35.55^c^
LWQ	143.90 ± 30.94^c^	160.53 ± 21.20^d^
Control	85.63 ± 10.00^d^	93.06 ± 13.26^e^

*The different letters within each column indicate statistically significant differences (p < 0.05).*

### Analysis of host monoterpenoids

**FIGURE 2:**
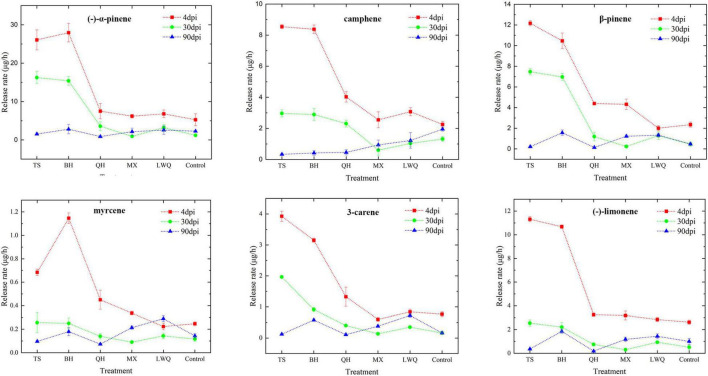
Release rates of six monoterpenes in different treatments (μg/h, mean ± SE, *N* = 3).

Due to the unavailability of α-phellandrene and γ-terpinene for detection, we analyzed and quantified the release rates of six different monoterpenes in whole seedlings, including (-)-α-pinene, camphene, β-pinene, myrcene, 3-carene, and (-)-limonene. From 4 to 90 dpi, the monoterpenoid release rate of spruce was the highest at 4 dpi and decreased gradually thereafter as shown in Figure 2. At 4 dpi, the release rates of monoterpenoids were significantly different between various strain treatments, particularly between the strains that were highly pathogenic strains and those that were the lowly pathogenic. For example, the six monoterpenes release rate in TS treatment were significantly higher than those in LWQ and control treatment (Figure 2; Supplementary Table 2). After 30 dpi, the release rates of the six monoterpenes among the inoculation treatments varied marginally. For example, at 90 dpi, the release rates of (-)-α-pinene, β-pinene, and myrcene were almost the same under the treatment of five strains, and same as the control as well (Figure 2; Supplementary Table 2). In addition, the release rates of different monoterpenes during the three time periods of the control treatments showed no significant differences (Figure 2; Supplementary Table 2). These results indicated that the changes in monoterpenoid release rates were caused by fungal infection, and not by wounding.

The release rate of monoterpenoids among five strain treatments showed a similar trend with the virulence gradients of five strains. In order to further analyze the relationship between monoterpenoids release rate and fungal infection ability, we conducted correlation analysis for the pathogenicity and monoterpenoid release rate at 30 dpi. As shown in Table 3, (-)-α-pinene, β-pinene, 3-carene, and (-)-limonene release rate were highly correlated with fungi pathogenicity (Pearson correlation analysis, *R*-value ≥ 0.919, *p* ≤ 0.01). Therefore, the amount of monoterpenoids released can be used to indicate the virulence of fungal strains.

**TABLE 3:** Correlation analysis for the pathogenicity and monoterpenoid changes at 30 dpi.

Pearson correlation with lesion	(-)-α -pinene	camphene	β -pinene	myrcene	3-carene	(-)-limonene
R	0.921	0.792	0.919	0.89	0.97	0.922
p	0.009	0.061	0.010	0.017	0.001	0.009

### Global review of transcriptome sequencing data

**FIGURE 3:**
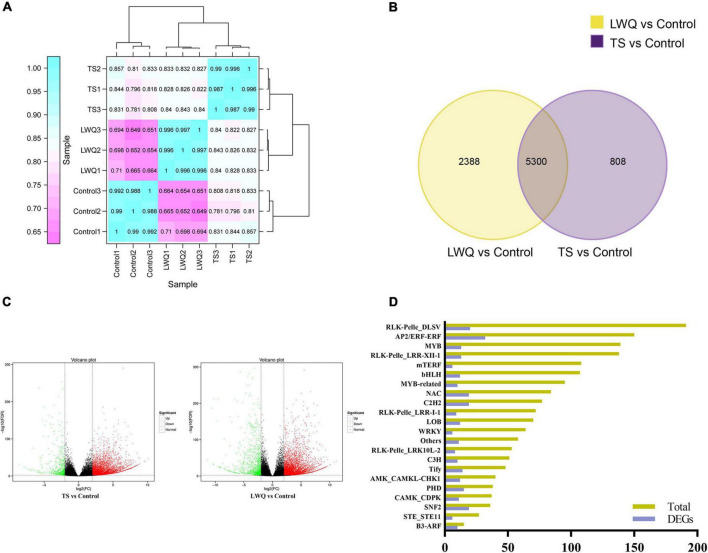
Global evaluation of transcriptome sequencing data of spruce. **(A)** Hierarchical clustering analysis of gene expression shown the correlation among samples. **(B)** Venn diagram of DEGs in compared between TS, LWQ, and control treatments. DEGs were selected using fold change ≥ 4, FDR correction < 0.01. **(C)** Volcano plot of all detected genes. Red represents upregulation; green represents downregulation; black represents non-differentially expression. **(D)** TFs differentially expressed under *O. bicolor*. *X*-axis represents the number of DEGs, and *Y*-axis represents the number names of transcription factor family.

Based on the results of lesion area and monoterpenoid release analyses, TS, LWQ, and control samples were selected for transcriptomic analysis, of which TS was a highly virulent strain and LWQ was a weakly virulent one. A total of 56.97 Gb of clean reads with Q30 > 93.57% were obtained from nine RNA-seq samples. The clean reads of each sample were aligned with the *Picea abies* reference genome (GCA_900067695.1), with efficiencies ranging from 79.23 to 80.55%, indicating high-quality raw data (Table 4). In total, 37,965 functionally annotated genes were identified (Supplementary Figure 1). A total of 24,433 new genes were discovered in all samples, of which 14,227 were sequenced and annotated to supplement and improve the original genome annotation (Supplementary Table 3). Pearson clustering analysis of the gene expression results showed that all the biological repeats in TS, LWQ, and control were clustered together (Figure 3A). The Pearson coefficient of all three biological replications in all treatments were ≥ 0.987, indicating that our sequencing data had high reliability (Figure 3A).

**TABLE 4:** Overview of the transcriptome sequencing dataset and quality check.

Sample ID	Read sum number	Base sum number	Q30 (%)	Total reads	Mapped reads (%)	Multi map reads
TS-1	20,136,203	6,021,668,790	94.88	40,272,406	32,438,452 (80.55%)	1,184,305 (2.94%)
TS-2	19,544,543	5,847,438,026	94.97	39,089,086	31,342,859 (80.18%)	1,153,232 (2.95%)
TS-3	20,900,628	6,253,541,488	94.42	41,801,256	33,512,254 (80.17%)	1,215,744 (2.91%)
LWQ-1	23,102,071	6,911,402,140	94.59	46,204,142	36,608,310 (79.23%)	1,368,647 (2.96%)
LWQ-2	20,447,827	6,115,226,536	94.77	40,895,654	32,782,193 (80.16%)	1,201,668 (2.94%)
LWQ-3	19,736,086	5,907,192,642	93.57	39,472,172	31,608,465 (80.08%)	1,152,093 (2.92%)
Control-1	21,604,871	6,463,536,296	94.43	43,209,742	34,288,798 (79.35%)	1,325,794 (3.07%)
Control-2	21,684,312	6,483,121,640	94.36	43,368,624	34,407,197 (79.34%)	1,318,281 (3.04%)
Control-3	23,305,398	6,970,566,612	94.31	46,610,796	37,068,186 (79.53%)	1,411,672 (3.03%)

### Differentially expressed genes in spruce infected by *Ophiostoma bicolor*

To explore the difference in the gene expression of *P. koraiensis* in response to various *O. bicolor* strains with different virulence, the transcriptomes of the seedlings in the TS, LWQ, and control treatment groups were compared. DEG parameters were set as follows: fold change ≥ 4 and FDR correction < 0.01. A total of 8,496 DEGs were detected in the two comparisons (TS vs. control, and LWQ vs. control), of which 5,300 DEGs were detected in the comparison of TS vs. control and LWQ vs. control, simultaneously (Figure 3B). In addition, 2,388 and 808 DEGs were detected in the comparison of TS vs. control and LWQ vs. control, respectively (Figure 3B). The number of upregulated genes accounted for more than 81.63% of the total DEGs, indicating that *O. bicolor* inoculation increased the overall gene expression of spruce at the genomic level (Figure 3C; Table 5, chi-square test, *p*-value < 0.0001). Annotation analysis suggested that the DEGs comprised many transcription factors (TFs), including AP2, RLK, NAC, C2H2, SNF2, PHD, and WRKY (Figure 3D; Supplementary Table 4). Compared to control, in TS- and LWQ-inoculated spruces, 281 and 353 TF-encoding genes were differentially expressed, among which 251 and 271 genes were upregulated (Table 5).

**TABLE 5:** Statistical of differentially expressed gene (DEG).

DEG Set	DEG Number	Upregulated	Downregulated	Ratio of up/down	Percentage of upregulated genes in total (%)	Upregulated TFs	Downregulated TFs	Percentage of upregulated genes in total (%)
TS vs. Control	6,108	5,464	644	8.48**	89.46	251	30	89.32
LWQ vs. Control	7,688	6,276	1,412	4.44**	81.63	271	82	76.77

The DEGs were selected using fold change ≥ 4, FDR correction < 0.01.Asterisks indicate the number of upregulated genes is more than that of downregulated genes (chi-square test, null hypothesis is Nd = Nu; **p < 0.0001).

Gene Ontology enrichment analysis was used to assign the DEGs to 46 GO terms. In biological processes, DEGs involved in cellular, metabolite, and signal-organism processes were enriched (Supplementary Figure 2). In terms of molecular function, the DEGs involved in regulated binding, catalytic activity, and transporter activity were enriched (Supplementary Figure 2). In the cellular component category, DEGs involved in cells, organelles, and membranes were enriched (Supplementary Figure 2). These results suggested that binding activity and high enzymatic activity were involved in the defense response of *P. koraiensis*.

**FIGURE 4:**
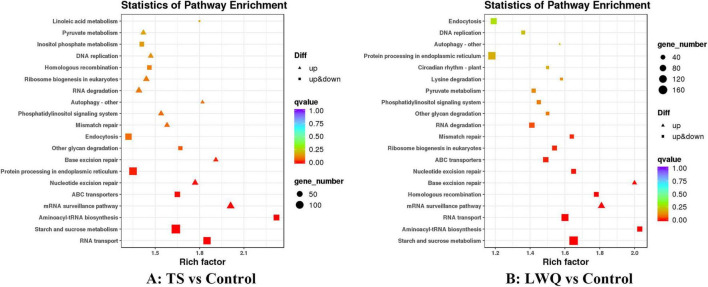
Top 20 KEGG pathway analysis of DEGs.

Kyoto encyclopedia of genes and genomes (KEGG) analysis enriched these DEGs in 13 metabolic pathways in all treatments (*q* < 0.05) (Figure 4; Supplementary Table 5; Kanehisa et al., 2021). Some of the *P. koraiensis* DEGs were mutually enriched in the TS and LWQ treatments, including RNA transport, starch and sucrose metabolism, aminoacyl-tRNA biosynthesis, mRNA surveillance pathway, ABC transporters, nucleotide excision repair, and base excision repair. Nevertheless, some DEGs were enriched only in one of the treatments. For example, DEGs involved in protein processing in the endoplasmic reticulum and other glycan degradation were enriched in the TS treatment, whereas DEGs involved in homologous recombination, ribosome biogenesis in eukaryotes, mismatch repair, and RNA degradation were enriched in the LWQ treatment (Figure 4 and Supplementary Table 5). DEGs involved in the mRNA surveillance and base excision repair pathways were upregulated, and DEGs in the remaining pathways were both upregulated and downregulated.

### Differentially expressed genes involved in terpenoid biosynthesis

**FIGURE 5:**
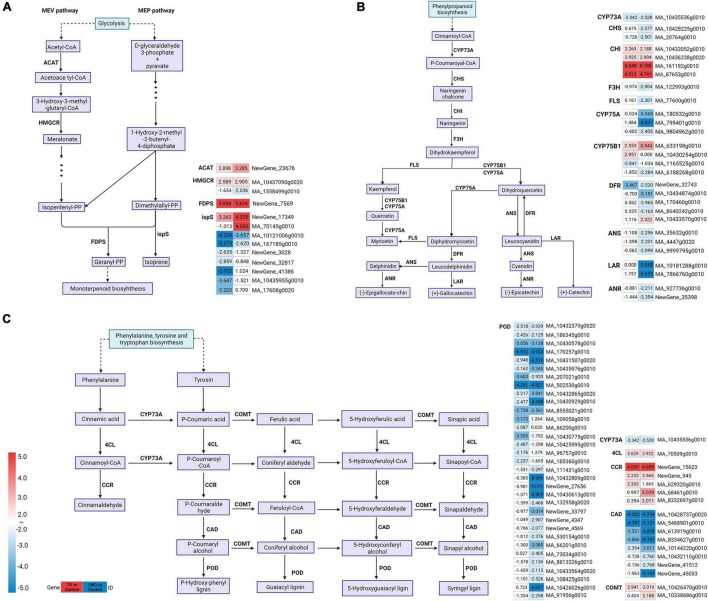
Transcriptional profiling of differentially expressed genes (DEGs) associated with Terpenoid backbone biosynthesis, flavonoid biosynthesis and phenylpropanoid biosynthesis pathway. The log_2_FC (fold change) values for the DEGs were used for each treatment (TS vs. Control and LWQ vs. Control). The progression of the color scale from blue to red represents an increase in the log_2_FC values. Sequences of all new genes were in Supplementary Table 7. **(A)** The DEGs involved in terpenoid backbone biosynthesis pathway. **(B)** The DEGs involved in flavonoid biosynthesis pathway. **(C)** The DEGs involved in phenylpropanoid biosynthesis pathway.

A total of 43 DEGs (24 upregulated and 19 downregulated) involved in terpenoid biosynthesis and metabolism were detected in TS vs. control and LWQ vs. control together (Supplementary Table 6). In both comparisons, from the viewpoint of terpenoid backbone biosynthesis pathway, key enzymes including acetyl-CoA C-acetyltransferase (ACAT) and hydroxymethylglutaryl-CoA reductase (HMGCR) involved in the isopentenyl pyrophosphate (IPP) synthesis, 4-hydroxy-3-methylbut-2-en-1-yl diphosphate reductase involved in the synthesis of dimethylallyl pyrophosphate (DMAPP), and farnesyl diphosphate synthase (FDPS) involved in the synthesis of monoterpenoid precursors geranyl pyrophosphate (GPP) from DMAPP, were activated, whereas isoprene synthase (ispS) involved in the synthesis of isoprene from DMAPP, were downregulated (Figure 5A; Supplementary Table 6). Enzyme genes in the monoterpene synthesis pathway were nearly unchanged, while ent-copalyl diphosphate synthase (ent-CPS) and miltiradiene synthase/copalyl diphosphate synthase (MDS) genes involved in the diterpenoid synthesis pathway were upregulated (Supplementary Table 6).

The DEGs related to terpenoid backbone biosynthesis were not identical in these two comparisons (Supplementary Table 6). For example, in LWQ treatment, the MA_1558499g0010 gene related to HMGCR synthesis was downregulated and MA_70145g0010 gene related to ispS synthesis was upregulated, while these two genes were not differentially expressed in TS treatment. In TS treatment the ispS synthesis related genes, Picea_abies_newGene_3028, Picea_abies_newGene_32817, Picea_abies_newGene_41386, MA_10435955g0010, and MA_17608g0020 genes were downregulated, but they were not differentially expressed in LWQ treatment.

### Differentially expressed genes involved in plant-pathogen interaction and mitogen-activated protein kinase signaling pathway

Of the 241 DEGs in the plant-pathogen interaction pathway, 212 DEGs were upregulated and involved in all changed nodes except pathogenesis-related protein 1 (PR1) node (Supplementary Figure 3; Supplementary Table 6). In TS vs. control, TS treatment activated the nodes of enhanced disease susceptibility 1 protein (EDS1) and elongation factor Tu (elf18). In LWQ vs. control, LWQ treatment activated the nodes of the molecular chaperone HtpG (HSP90A) and basic helix-loop-helix TF Upa20 (UPA20). Notably, the expression of calcium-binding protein (CML) nodes was activated by TS treatment and inhibited by LWQ treatment (Supplementary Table 6).

In mitogen-activated protein kinase (MAPK) signaling pathway, most of the 109 DEGs at 26 nodes were upregulated (Supplementary Figure 4; Supplementary Table 6). In addition to PR1, the catalase 1 (CAT1) gene also showed downregulated expression. Compared with TS vs. control, LWQ vs. control showed more downregulated DEGs in the MAPK signaling pathway, including basic endochitinase B (ChiB), ethylene-responsive TF 1 (ERF1), serine/threonine-protein kinase (OXI1), protein phosphatase 2C (PP2C), and vegetative storage protein 2 (VSP2).

### Differentially expressed genes involved in flavonoid and phenylpropanoid biosynthesis

In samples treated with the two *O. bicolor* strains, enzymes in the flavonoid biosynthesis pathway displayed various expression patterns. Chalcone isomerase (CHI) showed upregulated expression, *trans*-cinnamate 4-monooxygenase (CYP73A) showed downregulated expression, and flavonoid 3′-monooxygenase (CYP75B1) and bifunctional dihydroflavonol 4-reductase/flavanone 4-reductase (DFR) were both upregulated and downregulated (Figure 5B; Supplementary Table 6). In addition, in the samples inoculated with the low-virulence strain LWQ, the DEGs that encoded chalcone synthase (CHS), flavonoid 3′,5′-hydroxylase (CYP75A), naringenin 3-dioxygenase (F3H), flavonol synthase (FLS), anthocyanidin synthase (ANS), leucoanthocyanidin reductase (LAR), and anthocyanidin reductase (ANR) were downregulated, whereas these DEGs were not detected in the samples that were inoculated with high-virulence strain TS (Supplementary Table 6).

The results also showed that *O. bicolor* inoculation altered the expression of most genes encoding enzymes related to phenylpropanoid biosynthesis in *P. koraiensis* (Figure 5C). For example, the expression of genes encoding 4-coumarate CoA ligase (4CL), caffeic acid 3-O-methyltransferase/acetylserotonin O-methyltransferase (COMT), and cinnamoyl-CoA reductase (CCR) was upregulated. Genes encoding cinnamyl-CoA dehydrogenase (CAD), peroxidase (POD), and CYP73A were also downregulated. However, the genes encoding scopoletin glucosyltransferase (TOGT1) and beta-glucosidase (bglx) were both upregulated and downregulated. While some DEGs of *P. koraiensis* were enriched in the TS and LWQ treatment groups, some were enriched in either TS or LWQ treatment groups. For example, genes encoding coniferyl-aldehyde dehydrogenase (REF1) were solely enriched and downregulated with TS treatment, whereas genes encoding caffeoylshikimate esterase (CSE) were only enriched and upregulated with LWQ treatment (Supplementary Table 6).

### RT-qPCR

**FIGURE 6:**
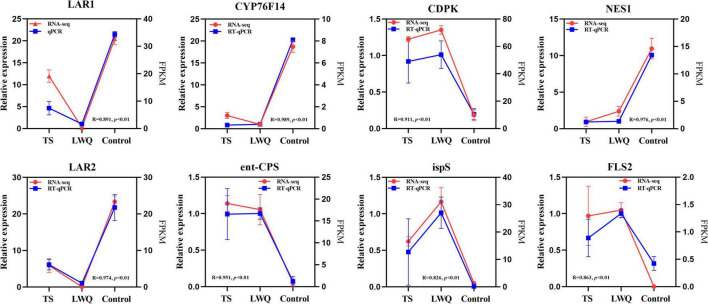
Validation of RNA-seq data by RT-qPCR. R was the correlation coefficient between the FPKM value of RNA-seq and the relative expression level of RT-qPCR, and *p* was the significance between the FPKM value of RNA-seq and the relative expression level of RT-qPCR. *p* < 0.01 means a significant correlation at the 0.01 level.

This study verified the expression of eight genes including calcium-dependent protein kinase (CDPK), ent-CPS, ispS, FLS2, LAR1, (E)-8-carboxylinalool synthase (CYP76F14), (3S,6E)-nerolidol synthase (NES1), and LAR2. Although the expression level of each gene in the transcriptome was higher than that of each gene in the RT-qPCR, both expression levels possessed consistent upregulated or downregulation as shown in Figure 6.

## Discussion

With global climate change, forest disasters caused by parasitic and semi-parasitic fungi have become more severe, which was also the case with the beetle-vectored fungi (Kim et al., 2021; Klesse et al., 2021; Morrison et al., 2021; Sitz et al., 2021; Li et al., 2022). However, compared with the former, the diseases caused by beetle-vectored fungi, particularly in gymnosperms, are not well studied. This study combined pathophysiological and transcriptomic analyses to investigate the defense response of *P. koraiensis* to *O. bicolor* infection. The results showed that: (1) different *O. bicolor* strains showed pathogenicity differentiation in *P. koraiensis*; (2) *O. bicolor* can induce the release of monoterpenoids in spruce in a short period of time; the release rates are correlated with the fungal virulence. Subsequently, the release rates gradually decrease to a normal level comparable to the control; (3) *O. bicolor* can significantly change the overall gene expression of *P. koraiensis* in early infection, and induce the upregulated expression of terpenoid backbone biosynthesis, plant-pathogen interaction, and MAPK signaling pathway.

Some ophiostomatoid fungi associated with *I. typographus* were pathogenic to spruce, and could induce the host to produce terpenoids, phenols, and other defense substances that were related to disease resistance (Solheim and Safranyik, 1997; Yamaoka et al., 2000; Sallé et al., 2005; Repe et al., 2015). In inoculation experiments by ophiostomatoid fungi, fungal virulence to hosts was not necessarily fatal (Kolb et al., 2019; Stewart et al., 2020). In this study, all five strains of *O. bicolor* could cause lesions around the inoculation site in *P. koraiensis* but could not cause wilting or death of the host tree (Figure 1). The elicited lesion areas around the inoculation sites decreased in the order of TS, BH, QH, MX, and LWQ (Table 2). Devkota and Eckhardt (2018) showed that there were differences in host defense responses to different fungal strains inoculation. These results indicated that *O. bicolor* was a fungus that was weakly virulent to *P. koraiensis*, and there was pathogenicity differentiation among different strains.

Trees can generate and transfer terpenes, such as monoterpenes, diterpenes, and sesquiterpenes, which are toxic to beetles and fungi; these terpenes could prevent further attacks from beetles and fungi. Raffa and Berryman (1983) reported that lodgepole pines could resist mountain pine beetles by producing high concentrations of two monoterpenes, α-pinene, and limonene, which was further verified in the field by Erbilgin et al. (2017). Meanwhile, the resistance induced by the bark beetle in the host also inhibits the infection of the fungus (Lombardero et al., 2019). The results of this study showed that in addition to the pathogenicity differentiation of different fungi strains, the spruce monoterpenoids synthesis that was induced by *O. bicolor*, also showed differentiation (Supplementary Table 2). Furthermore, the release rate of monoterpenoids from the host increased at first and peaked at 4 dpi. From 4 to 90 dpi, the release rate of monoterpenoids showed a gradual downward trend (Figure 2). The release rates of monoterpenoids were correlated with the fungal virulence at 30 dpi and almost did not differ with each other under all treatments at 90 dpi. This phenomenon is consistent with the findings of a previous study, which showed that after MeJA was applied to *P. abies*, the host displayed an induction of terpenes, with concentrations peaking around 16 days after treatment and returning to near-normal levels within 32 days after treatment (Erbilgin et al., 2006). Mageroy et al. (2020a) further verified this phenomenon in Norway spruce, which might be the result of defense priming. Previous studies have shown that host trees inoculated with ophiostomatoid fungi, i.e., *E. polonica* and *Endoconidiophora fujiensis*, could generate a rapid and long-term release of monoterpenoids, which possibly deplete the host defense ultimately (Arango-Velez et al., 2018; Fang et al., 2020; Ott et al., 2021). In this study, the release of monoterpenoids did not drastically increase, which was likely due to the different fungal species and inoculation scenarios. The dominant species of ophiostomatoid fungi, which is less virulent, was selected to evaluate host response, whereas in the previous study, the pioneer species, which is more virulent, was selected. This result is in accordance with the conclusion that more virulent fungi induce large changes in host defense terpenoids (Cale et al., 2019; Fang et al., 2020). Therefore, we speculate that *O. bicolor* stimulates the host to produce defense priming; however, subsequent experiments using MeJA treatment as a positive control of priming defense and challenging stimulus, e.g., *E. polonica* inoculation or beetle infestation are needed for further verification.

To reveal the regulation of gene expression related to disease resistance and immunity in *P. koraiensis*, transcriptomic analysis was performed. The results showed that in the early stage of *O. bicolor* infection of spruce stems (4 dpi), most of the DEGs were upregulated (Figure 3), and these DEGs were mainly related to repair, binding, and signal transduction processes. In the transcriptomic analysis of *Grosmannia clavigera* hyphae treated with terpenoids for 12 h, it was observed that genes encoding DNA repair, recombination, stabilization, and replication proteins were induced (DiGuistini et al., 2011). This study expands our knowledge of the effects of ophiostomatoid fungi on hosts. Early defense signals in plants often lead to the activation of downstream TF genes, thereby enhancing the expression of defense-related genes (Eulgem and Somssich, 2007). WRKYs play important roles in plant immunity through the ET, JA, and SA pathways in response to various biotic stressors. Several WRKY TFs (mostly upregulated) were involved in the spruce response to *O. bicolor*. We focused on previously reported pathways involved in plant immune responses, such as plant-pathogen interactions, MAPK signaling, flavonoid biosynthesis, phenylpropane biosynthesis, and terpene biosynthesis and metabolism, to elucidate the molecular mechanisms underlying host defense responses (Gao et al., 2018; Su et al., 2018; Song et al., 2019).

In plants, the biosynthetic precursor of terpenoids can be synthesized via two pathways: the MVA and MEP pathways (Roberts, 2007). In this study, numerous DEGs involved in terpenoid biosynthesis by the MVA and MEP pathways were upregulated (Figure 5A; Supplementary Table 6). On the one hand, the genes of FDPS, which are involved in the synthesis of various monoterpene precursors, and GPP, which comes from DMAPP, were upregulated. On the other hand, the genes of ispS involved in the synthesis of isoprene from DMAPP, were downregulated and thus enhanced the monoterpenoids precursor biosynthesis (Figure 5A). These results suggest that *O. bicolor* inoculation in *P. koraiensis* promoted the biosynthesis of host monoterpene precursor, thereby increasing the release of six monoterpenoids. The expression of enzyme genes in the monoterpene synthesis pathway remained nearly unchanged, which is expected and consistent with the findings of previous research (Mageroy et al., 2020b). Moreover, the differences of DEGs related to ispS and HMGCR in TS and LWQ treatments may be the reason for the differences in the release rates of monoterpenoids in the two strain treatments.

*Ophiostoma bicolor* significantly altered the expression of genes related to plant-pathogen interactions pathways, involving 241 DEGs at 25 nodes. Upregulated genes accounted for 87.97% and were mainly involved in biological processes such as hypersensitive response (HR), cell wall reinforcement, stomatal closure, defense-related gene induction, phytoalexin accumulation, miRNA production, programmed cell death, suppression of plant HR, and defense response (Supplementary Figure 3). These processes are closely related to plant defense responses (Yang et al., 1997). Binding of plant PR proteins to pathogen effector proteins activates ion channels, oxidative bursts, and other signal transductions (Dixon et al., 1994). Ca^2+^ activates the plant early surveillance system to prevent microbial infection in plant defense signaling (Nürnberger and Scheel, 2001). In the present study, the host treated with the two strains (TS and LWQ treatments) showed differential expression pattern of CML, and LWQ treatment showed less downstream calcium-dependent process in defense response.

The upregulated genes in the MAPK signaling pathway accounted for 68.81% (75 of 109) of all upregulated genes and were mainly involved in biological processes such as cell death, accumulation of reactive oxygen species in plant defense, defense responses, stress adaptation, and wounding. The downregulated genes were mainly related to defense responses against pathogens and wounding (Supplementary Figure 4). MAPK cascades play an important role in plant defense against pathogens (Pitzschke et al., 2009). Its activation is one of the earliest responses for sensing pathogen-associated molecular patterns (PAMPs) in plants (Meng and Zhang, 2013). Some DEGs of LWQ treatment were downregulated and enriched in defense response against pathogens, cell death process and active oxygen production process, while these genes were not differentially expressed in TS treatment (Supplementary Table 6), which might indicate weaker induction of host defense response by the weakly virulent strain. These results suggest that *P. koraiensis* may use different MAPKs to deliver attack signals, and *O. bicolor* activated the earliest defense via the MAPK cascade, thus inhibiting further infection.

Through the phenylpropanoid pathway, many kinds of lignin (p-hydroxyphenyl lignin, guaiacyl lignin, 5-hydroxy-guaiacly lignin, and syringyl lignin) can be produced by plants. Lignin can help plants resist biotic and abiotic stresses by regulating secondary cell wall development and stomata (Chen et al., 2019). The synthesis process can be roughly divided into two steps: firstly, the lignin monomer is synthesized under catalysis by a series of enzymes including the 4CL and CAD, and secondly the lignin monomer is polymerized into bioactive lignin by a series of chemical reactions catalyzed by POD and other enzymes (Boerjan et al., 2003). Inhibition of CAD activity in vascular plants changed lignin content. For example, a decrease in CAD activity in sorghum results in a decrease in the total amount of lignin (Palmer et al., 2008). In the present study, the key enzymes involved in lignin monomer synthesis, including 4CL, COMT, and CCR, were activated (Supplementary Table 6). However, CAD required for the final step of lignin monomer biosynthesis was inhibited (Supplementary Table 6). In addition, the gene expression of POD was downregulated, which is consistent with the findings of previous studies on poplar responses to canker pathogens (Li et al., 2019). Although our experiment did not measure the change in lignin content in spruce, it was known from the literature that the content of lignin was reduced in hosts infected with ophiostomatoid fungi (Villari et al., 2012). As a result, *O. bicolor* may inhibit the synthesis and metabolism of lignin in the spruce stem.

The flavonoid biosynthesis pathway produces a variety of phenolic compounds such as procyanidin, catechin, gallocatechin, and epicatechin. When pathogens invade spruce, fluorescent inclusion bodies containing phenolic compounds appear in the phloem parenchyma cells of the bark, suggesting that phenolic compounds may play a key role in defense against herbivores and pathogens (Franceschi et al., 2005). After an *E. polonica* infection in Norway spruce, the content of flavane-3-ols, catechin, and gallocatechin in the bark increased (Hammerbacher et al., 2014). The results of this study showed that most DEGs (22 of 28) associated with the flavonoid biosynthesis pathway were downregulated at 4 dpi (Supplementary Table 6), and *O. bicolor* infection inhibited phenolics-related gene expression in spruce, which differed from the expression pattern of the virulent pioneer *E. polonica*. The downregulated expression pattern in this study was consistent with the transcriptomic results of Norway spruce infected with *Chrysomyxa rhododendri* at 4 dpi (Trujillo-Moya et al., 2020). The flavonoid contents of spruces inoculated by *O. bicolor* were not investigated in this study. Thus, further research is needed to clarify the correlation between the contents and gene expressions of flavonoids.

## Conclusion

The ophiostomatoid fungal flora of bark beetles is rich and diverse. This study showed that a dominant fungus of the flora, *O. bicolor*, is a weakly virulent pathogen of spruce, and various strains showed differentiation in pathogenicity. The release of monoterpenoids from the host is positively correlated with the virulence of the inoculated fungus. In terms of amounts of monoterpenoids released by hosts after inoculation, the pioneer species, e.g., *Endoconidiophora* spp., can induce rapid and long-term defense responses in the host, while *O. bicolor* is likely to induce the host defense priming phenomenon. The response of spruce to *O. bicolor* is a complex process involving multiple biological processes, including plant-pathogen interaction process and MAPK metabolic process. The expression patterns of spruce defense-related genes during infection with different *O. bicolor* strains were different. However, it was still unclear how the host coordinates these different defense responses against different fungal strains. Further studies are necessary to investigate the underlying mechanism of the host defense response against the pathogen fungi. Bark beetles and their associated fungi usually attack adult trees, thus our inoculations conducted on seedlings have certain limitations. The experiments should attempt to conduct under natural conditions in the future.

## Data Availability Statement

The datasets presented in this study can be found in online repositories. The names of the repository/repositories and accession number(s) can be found in the article/Supplementary Material.

## Author contributions

QL and YL conceptualized and designed the research. YL, QZ, and GZ performed the experiments. YL and QZ analyzed the data and wrote the manuscript. YL, QZ, ZW, HW, JZ, and QL participated in the discussion for experimental details. QL and JZ revised the manuscript. QL directed the project. All authors contributed to the article and approved the submitted version.

## Conflict of Interest

The authors declare that the research was conducted in the absence of any commercial or financial relationships that could be construed as a potential conflict of interest.

## Publisher’s Note

All claims expressed in this article are solely those of the authors and do not necessarily represent those of their affiliated organizations, or those of the publisher, the editors and the reviewers. Any product that may be evaluated in this article, or claim that may be made by its manufacturer, is not guaranteed or endorsed by the publisher.

## Footnotes

1
www.biocloud.net

## Supplementary Material

Supplementary Figure 1 Functional annotation of the all genes detected in *P. koraiensis* from various databases. **(A)** NR database homologous species distribution analysis; **(B)** GO; **(C)** eggNOG; **(D)** KOG; and **(E)** COG.

Supplementary Figure 2 Gene Ontology (GO) classification of DEGs.

Supplementary Figure 3 Plant-pathogen interaction pathway map. Upregulation is highlighted in red; downregulation is highlighted in green; mixed regulation is highlighted in blue.

Supplementary Figure 4 Mitogen-activated protein kinase (MAPK) signaling pathway map. Upregulation is highlighted in red; downregulation is highlighted in green; mixed regulation is highlighted in blue.

Supplementary Table 1 Primer sequences used in RT-qPCR.

Supplementary Table 2 Release rates of major monoterpenes in different treatments (μg/h, mean ± SE, *N* = 3).

Supplementary Table 3 Statistics of annotation results for new genes in databases.

Supplementary Table 4 All the different transcription factors.

Supplementary Table 5 Statistics of KEGG pathway with significant enrichment.

Supplementary Table 6 Differential gene expression levels in five KEGG pathways.

Supplementary Table 7 Sequences of all differentially expressed new genes involved in five KEGG pathways.
