# Supplementary material for: Pathophysiology and transcriptomic analysis of Picea koraiensis inoculated by bark beetle-vectored fungus Ophiostoma bicolor
Source: Front Plant Sci. 2022 Jul 19;13:944336. doi: 10.3389/fpls.2022.944336 (PMC9345248; doi:10.3389/fpls.2022.944336)
Supplement: Supplementary Figure 1 — Functional annotation of the all genes detected in P. koraiensis from various databases. (A) NR database homologous species distribution analysis; (B) GO; (C) eggNOG; (D) KOG; and (E) COG. [file Data_Sheet_1.docx]

Supplementary Material

# Supplementary Figures and Tables

## Supplementary Figures


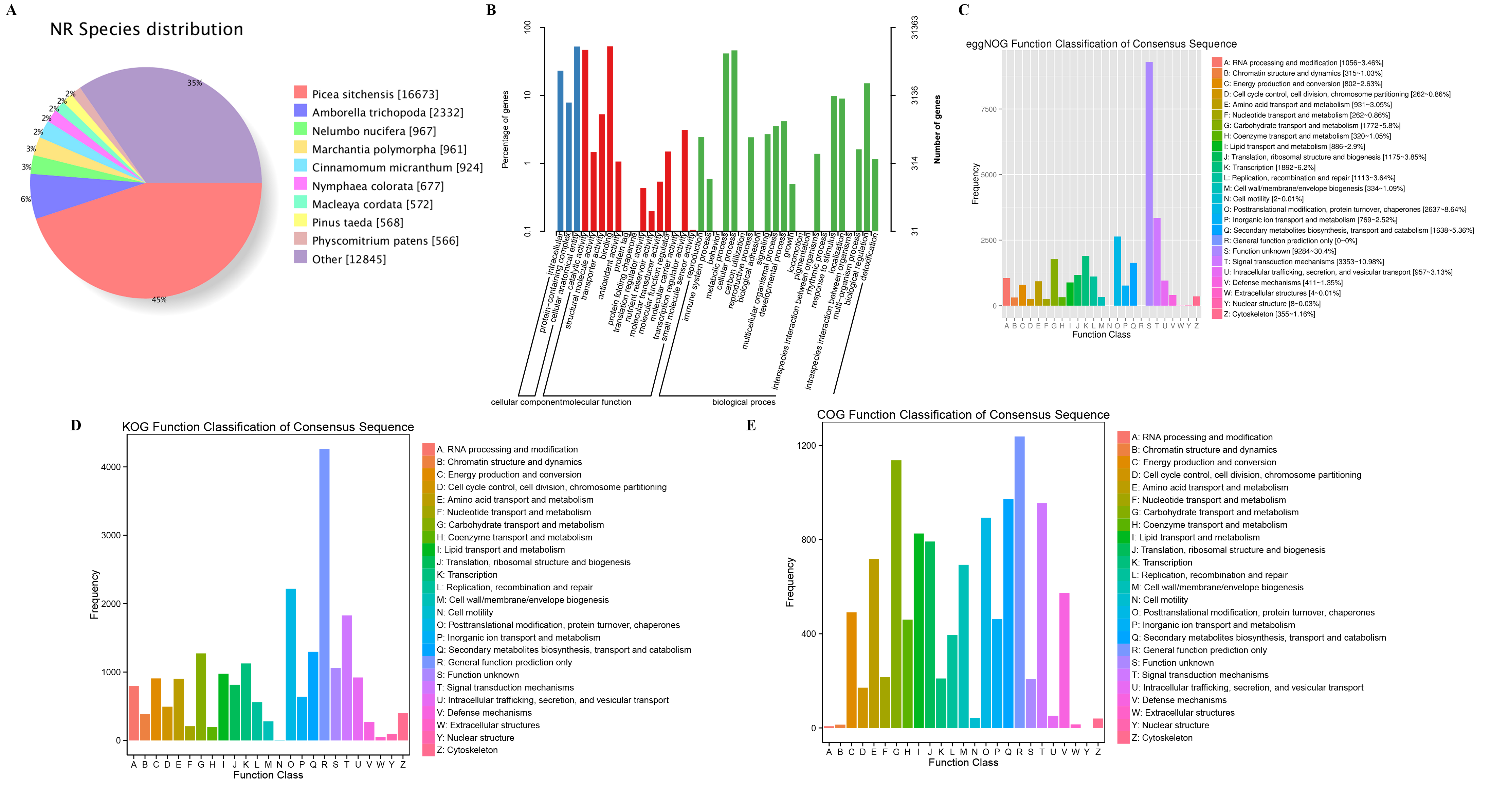
**Supplementary Figure 1.** Functional annotation of the all genes detected in P. koraiensis from various databases. (A) NR database homologous species distribution analysis; (B) GO; (C) eggNOG; (D) KOG; and (E) COG


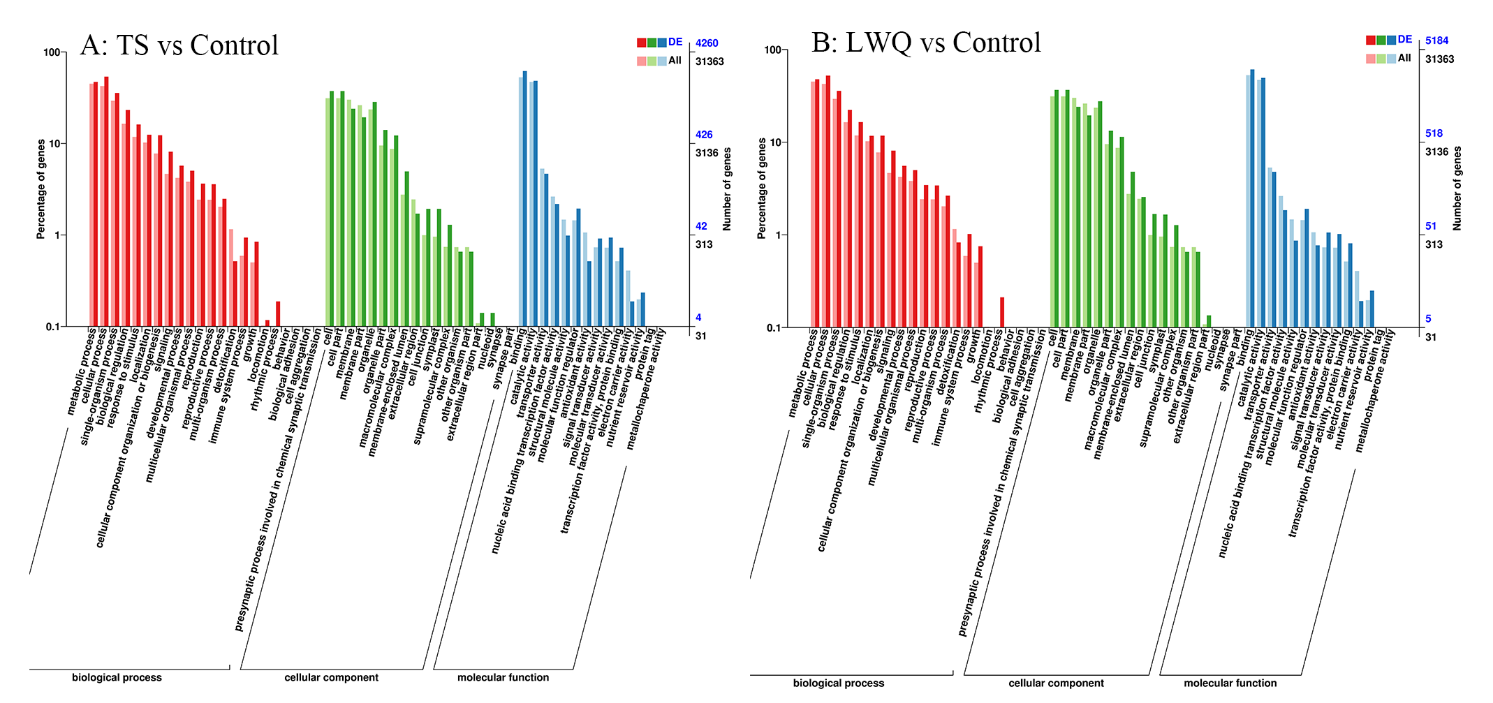


**Supplementary Figure 2** GO classification of DEGs


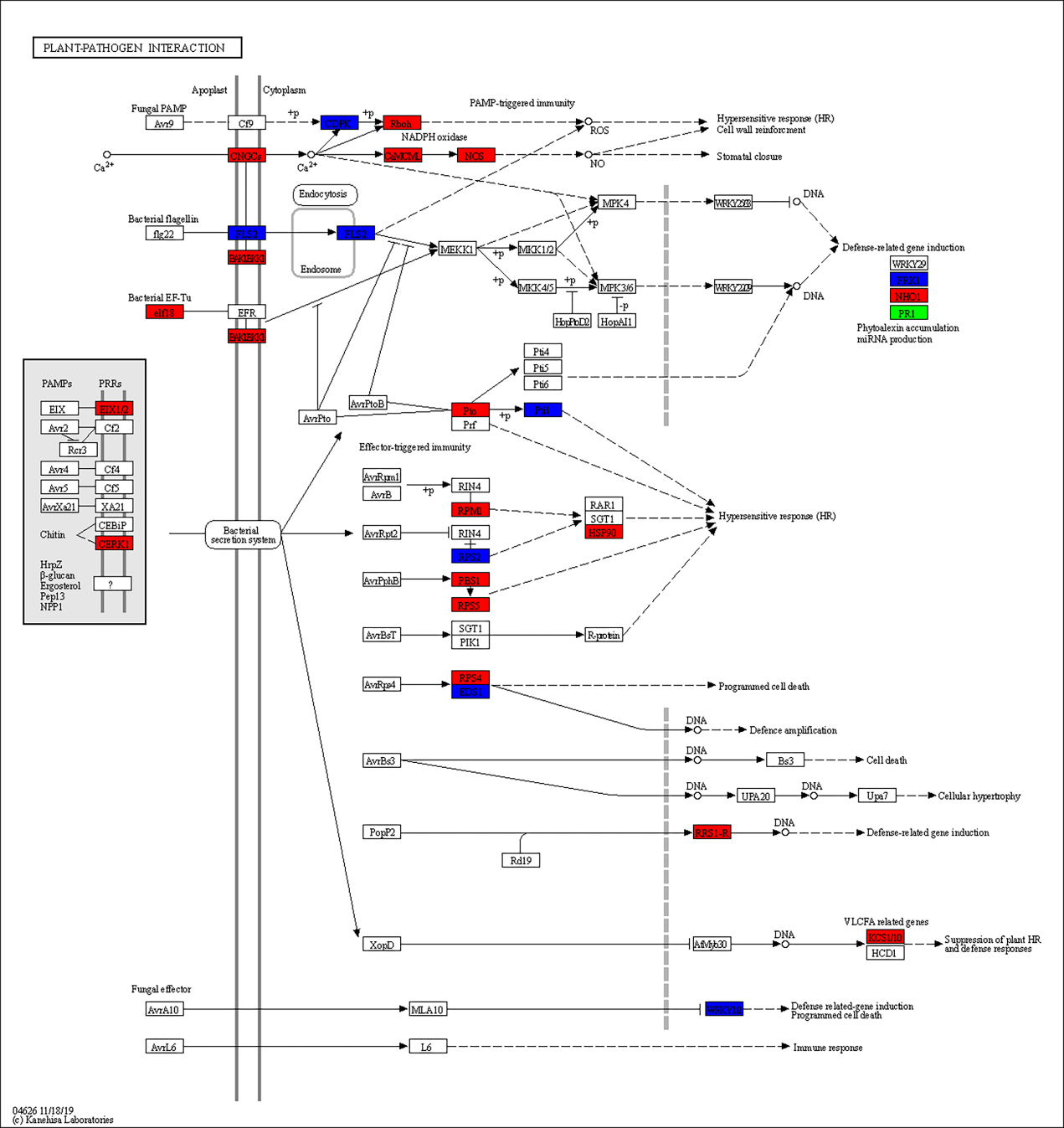


**Supplementary Figure 3** Plant-pathogen interaction pathway map. Upregulation is highlighted in red; downregulation is highlighted in green; mixed regulation is highlighted in blue.


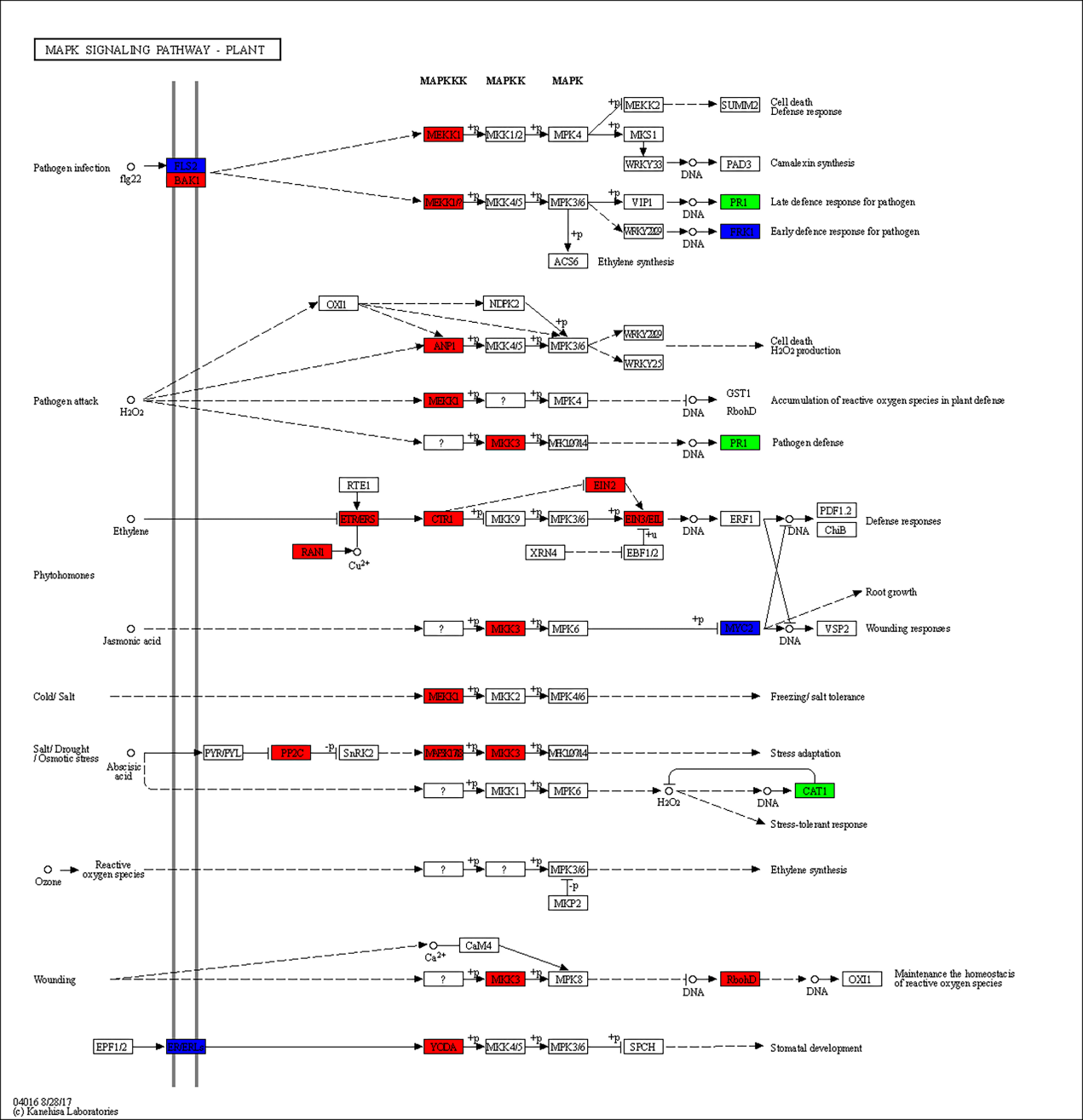


**Supplementary Figure 4** MAPK signaling pathway map. Upregulation is highlighted in red; downregulation is highlighted in green; mixed regulation is highlighted in blue.

## Supplementary Tables

**Supplementary Table 1:** Primer sequences used in qRT-PCR

| Number | Symbol | Gene ID | Primer (5'to3') |
| --- | --- | --- | --- |
| 1 | LAR1 | MA_10181288g0010F | CTGAAGTGGCTCATCCTGT |
|  |  | MA_10181288g0010R | TAACCTGTAGCTCCAATCA |
| 2 | CYP76F14 | MA_10432446g0020F | CAGGTGCTGGTAAATGTATG |
|  |  | MA_10432446g0020R | AGTCGAAGGAGTGGAGGAG |
| 3 | CDPK | MA_110181g0010F | AAAACCCAAGTTCAAATCC |
|  |  | MA_110181g0010R | GCCCAGTCTCCTACCCAAT |
| 4 | NES1 | MA_5874804g0010F | CCTGGATTAACAGAGGAAG |
|  |  | MA_5874804g0010R | ATTGTTGGAGACGGTGTAG |
| 5 | LAR2 | MA_7866760g0010F | CTGACATTGGCAAATACAC |
|  |  | MA_7866760g0010R | CTTGTTCCGAGATGCAGAC |
| 6 | ent-CPS | Picea_abies_newGene_16560F | ATGGGCTATGGGGAAACTA |
|  |  | Picea_abies_newGene_16560R | CATATGCCAAGAAGTAGAA |
| 7 | ispS | Picea_abies_newGene_17439F | ATGAATCTTCCATCCCTCG |
|  |  | Picea_abies_newGene_17439R | CAGAAAACTAAAAGCAAACA |
| 8 | FLS2 | Picea_abies_newGene_34364F | CAGGAGGCTTCTCGTTTAT |
|  |  | Picea_abies_newGene_34364R | ACATTGCTGGGCTTTACAT |
| Actin | AAF03692 | MA_17028g0010F | TGAGCTCCCTGATGGGCAGGTGA |
|  |  | MA_17028g0010R | TGGATACCAGCAGCTTCCATCCCAAT |

**Supplementary Table 2:** Release rates of major monoterpenes in different treatments (μg/h, mean±SE, N=3)

| **Period** | **Strain no.** | **(-)-****α-pinene** | **camphene** | **β-pinene** | **myrcene** | **3-carene** | **(-)-limonene** |
| --- | --- | --- | --- | --- | --- | --- | --- |
| 4dpi | TS | 26.06±2.60Aa | 8.55±0.13Aa | 12.26±0.25Aa | 0.68±0.03Ab | 3.93±0.16Aa | 11.31±0.22Aa |
|  | BH | 27.95±2.41Aa | 8.38±0.27Aa | 10.46±0.76Aa | 1.15±0.04Aa | 3.15±0.07Aa | 10.68±0.15Aa |
|  | QH | 7.49±2.01Ab | 4.03±0.34Ab | 4.40±0.13Ab | 0.45±0.08Ac | 1.33±0.31Ab | 3.25±0.14Ab |
|  | MX | 6.19±0.50Ab | 2.55±0.51Ab | 4.32±0.51Ab | 0.34±0.02Acd | 0.60±0.07Ac | 3.18±0.37Ab |
|  | LWQ | 6.79±1.00Ab | 3.07±0.26Ab | 2.00±0.24Ab | 0.22±0.03Ad | 0.84±0.08Ac | 2.85±0.18Ab |
|  | Control | 5.26±1.56Ab | 2.25±0.20Ab | 2.35±0.25Ab | 0.25±0.02Ad | 0.77±0.08Ac | 2.62±0.18Ab |
| 30dpi | TS | 16.25±1.56Ba | 2.97±0.23Ba | 7.48±0.31Ba | 0.26±0.08Ba | 1.96±0.03Aa | 2.55±0.27Ba |
|  | BH | 15.38±1.11Ba | 2.89±0.39Ba | 6.97±0.34Ba | 0.25±0.05Aa | 0.91±0.07Bb | 2.21±0.38Ba |
|  | QH | 3.58±1.15ABb | 2.31±0.22Ba | 1.19±0.41Bb | 0.14±0.02Bb | 0.40±0.02Bc | 0.75±0.12Bb |
|  | MX | 0.93±0.20Bb | 0.60±0.32Bc | 0.24±0.11Bbc | 0.19±0.01Bb | 0.14±0.04Bd | 0.31±0.09Bb |
|  | LWQ | 3.19±0.83Bb | 1.03±0.20Bbc | 1.30±0.37Abc | 0.14±0.02Bb | 0.35±0.04Bc | 0.93±0.12Bb |
|  | Control | 1.19±0.27Ab | 1.32±0.17Bb | 0.46±0.22Bc | 0.12±0.02Bb | 0.16±0.02Bd | 0.52±0.18Bb |
| 90dpi | TS | 1.53±0.29Ca | 0.33±0.07Cc | 0.21±0.03Aa | 0.10±0.00Ca | 0.12±0.02Bb | 0.36±0.03Cb |
|  | BH | 2.80±1.21Ca | 0.42±0.15Cb | 1.57±0.27Ca | 0.18±0.03Ba | 0.58±0.03Ca | 1.85±0.17Ba |
|  | QH | 0.88±0.19Ba | 0.46±0.17Cb | 0.14±0.01Ca | 0.07±0.00Ba | 0.11±0.01Cb | 0.19±0.03Cb |
|  | MX | 2.16±0.91Ba | 0.93±0.31Bab | 1.23±0.12Ba | 0.21±0.01Ca | 0.38±0.04Cab | 1.18±0.18Ca |
|  | LWQ | 2.57±1.11Ba | 1.22±0.50Bab | 1.33±0.19Aa | 0.29±0.03Aa | 0.73±0.06Aa | 1.44±0.21Aa |
|  | Control | 2.28±0.20Aa | 1.95±0.14ABa | 0.47±0.11Ba | 0.14±0.02Ba | 0.16±0.03Bb | 1.00±0.26Ba |

Note: Lower-case letters in a column in the same time period show results of significant differences between treatments and capital letters in a column in the same treatments show results of significant differences between the time points. The same letters within each column (capital letters and lower-case letters) indicate no statistically significant differences (*P*<0.05)

| **Annotated Database** | **Annotated Number** | **Annotated Percent** (%) |
| --- | --- | --- |
| NR | 13737 | 56.2 |
| SwissProt | 8616 | 35.3 |
| GO | 11978 | 49 |
| COG | 3098 | 12.7 |
| KOG | 7593 | 31.1 |
| EggNOG | 11219 | 45.9 |
| KEGG | 9115 | 37.3 |
| All | 14227 | 58.2 |

**Table Supplementary 3.** Statistics of annotation results for new genes in databases
